# Design and Preparation of Heterostructured Cu_2_O/TiO_2_ Materials for Photocatalytic Applications

**DOI:** 10.3390/molecules29215028

**Published:** 2024-10-24

**Authors:** Yating Tai, Boxuan Yang, Jing Li, Lingshi Meng, Pengcheng Xing, Shengjie Wang

**Affiliations:** College of Chemistry and Chemical Engineering, China University of Petroleum, Qingdao 266580, China; s22030165@s.upc.edu.cn (Y.T.); s23030148@s.upc.edu.cn (B.Y.); lijing@upc.edu.cn (J.L.); z24030168@s.upc.edu.cn (L.M.); z24030181@s.upc.edu.cn (P.X.)

**Keywords:** Cu_2_O/TiO_2_, heterojunction, photocatalysis, light absorption, mechanism of charge separation and transfer, application

## Abstract

The extensive use of fossil fuels has sped up the global development of the world economy and is accompanied by significant problems, such as energy shortages and environmental pollution. Solar energy, an inexhaustible and clean energy resource, has emerged as a promising sustainable alternative. Light irradiation can be transformed into electrical/chemical energy, which can be used to remove pollutants or transform contaminants into high-value-added chemicals through photocatalytic reactions. Therefore, photocatalysis is a promising strategy to overcome the increasing energy and environmental problems. As is well-known, photocatalysts are key components of photocatalytic systems. Among the widely investigated photocatalysts, titanium dioxide (TiO_2_) has attracted great attention owing to its excellent light-driven redox capability and photochemical stability. However, its poor solar light response and rapid recombination of electron–hole pairs limit its photocatalytic applications. Therefore, strategies to enhance the photocatalytic activity of TiO_2_ by narrowing its bandgap and inhibiting the recombination of charges have been widely accepted. Constructing heterojunctions with other components, including cuprous oxide (Cu_2_O), has especially narrowed the bandgap, providing a promising means of solving the present challenges. This paper reviews the advances in research on heterostructured Cu_2_O/TiO_2_ photocatalysts, such as their synthesis methods, mechanisms for the enhancement of photocatalytic performance, and their applications in hydrogen production, CO_2_ reduction, selective synthesis, and the degradation of pollutants. The mechanism of charge separation and transfer through the Cu_2_O/TiO_2_ heterojunctions and the inherent factors that lead to the enhancement of photocatalytic performance are extensively discussed. Additionally, the current challenges in and future perspectives on the use of heterostructured Cu_2_O/TiO_2_ photocatalysts are also highlighted.

## 1. Introduction

Human society has experienced unprecedentedly rapid development and prosperity following the widespread use of fossil fuels. However, the overuse of fossil fuels leads to continuous environmental deterioration and energy shortages. Therefore, it is imperative to find alternative sources of clean energy to replace nonrenewable fossil fuels. Among the new energy resources, solar energy has attracted significant attention owing to its abundant availability, geographic independence, and renewable nature. Developing efficient technologies for converting solar energy into chemical or electrical power provides a promising solution to the energy crisis and environmental issues [1,2]. Photocatalysis, a powerful technology that uses light energy to drive chemical reactions [3], has been widely used in hydrogen production [4,5,6,7], CO_2_ reduction [8,9,10,11,12,13], and pollutant degradation in air and water [14,15]. Additionally, it has promising applications in self-cleaning surfaces [16], as well as anti-fouling [17] and biomedical devices [18]. As is known, photocatalytic reactions rely on photocatalysts. Under light irradiation, photocatalysts generate electrons with high reduction potential, hydroxyl radicals [19], and reactive oxygen species [20] with strong oxidative capabilities [21], which enable photoreduction or photooxidation reactions to be conducted successfully. Photocatalysts can be classified into organic, inorganic, and organic–inorganic hybrid materials according to their composition [22,23,24,25,26,27,28,29]. Inorganic photocatalytic materials, including titanium dioxide (TiO_2_), cuprous oxide (Cu_2_O), and zinc oxide (ZnO) [30,31,32,33], exhibit excellent photostability and recyclability. Research on inorganic photocatalysts has made remarkable progress since Fujishima and Honda [34] discovered photocatalytic water-splitting over TiO_2_ electrodes in 1972.

Among the inorganic photocatalysts, TiO_2_ is considered one of the most representative [35,36] materials due to its strong redox capability, super-hydrophilic properties, high photochemical stability, non-toxicity, low cost, and accessibility [20,37,38,39,40,41,42]. However, certain inherent shortcomings of TiO_2_ have limited its further applications [43]. Due to its wide bandgap [44], it is sensitive only to ultraviolet light, not to visible light and near-infrared light, resulting in low efficiency regarding sunlight use. Furthermore, the fast recombination rate of the photogenerated electron–hole pairs [45] reduces its charge transfer efficiency and thus decreases its photocatalytic performance. Consequently, different techniques, including element/ion doping [36], the surface deposition of noble metals [46], constructing heterojunctions with other semiconductors [47], and dye sensitization [48], have been employed to enhance its visible light absorption and electron–hole separation efficiency, thereby improving its photocatalytic activity.

Doping technologies include non-metal doping (N [49,50,51], C [52,53,54], and S [55,56], etc.) and metal doping (Fe [57], Co [58,59], and Cr [60,61], etc.). Generally, non-metal doping can extend light absorption to the visible region [62,63] by elevating the valence band, while metal doping improves the separation and transfer rates of charge carriers. However, the doping process might cause lattice distortion and have an uneven distribution. Surface modification means introducing functional groups or organic molecules onto the surface of TiO_2_ materials to alter their surface properties and enhance their visible light absorption. Precise control is essential to avoid excessive impurities or insufficient coverage, and the photostability of the introduced organic units is also a challenge. Heterojunctions, formed by two or more semiconductors with different band structures, can effectively broaden the light response range and thus enhance solar energy utilization. Suitable heterojunctions formed between TiO_2_ and other semiconductors (e.g., Cu_2_O) can optimize the charge transfer paths and reduce their recombination rates. This will facilitate the efficient separation and transfer of charge carriers and thus lead to high reactivity under solar light irradiation.

Cuprous oxide (Cu_2_O) semiconductors have several significant advantages, such as a narrow band gap, excellent electrical conductivity, abundant resources, and environmental friendliness. This endows them with excellent visible light sensitivity and unique photocatalytic applications. However, pure Cu_2_O photocatalysts often suffer from the problems of photocorrosion and the rapid recombination of charge carriers. Heterojunctions formed with TiO_2_ can exhibit significantly improved photocatalytic performances due to the synergistic effects of spectral expansion, charge separation enhancements, and improved stability. Therefore, the design and construction of Cu_2_O/TiO_2_ heterojunctions is a key strategy to expand the application of photocatalysis and improve the efficiency of semiconductor photocatalysts.

In this paper, we summarized the characteristics and formation process of different Cu_2_O/TiO_2_ heterojunctions, analyzed their intrinsic mechanism for the enhancement of photocatalytic activity, and discussed their applications in hydrogen production, CO_2_ reduction, pollutant removal, and selective synthesis. Here, we pay more attention to the mechanism of charge separation and transfer through the Cu_2_O/TiO_2_ heterojunctions during the photocatalytic process. This can provide a broader understanding of the advances in research on heterostructured Cu_2_O/TiO_2_ materials and can provide creative ideas for the design of more efficient photocatalysts. Additionally, we summarized the challenges in the research and application of Cu_2_O/TiO_2_ heterojunctions and discussed future research perspectives.

## 2. Heterojunctions of Cu_2_O/TiO_2_ Materials

### 2.1. Heterojunction Type

The rapid recombination of photogenerated electrons and holes in the photocatalytic process and the limited oxidation or reduction capability of single materials result in unsatisfactory photocatalytic efficiency. Thus, various heterostructures were designed and constructed by combining different semiconductors [64,65]. The effect of the interfacial structure, the composition, and the intrinsic relationship between the heterostructures and photocatalytic performances was investigated systematically. The results provide solutions to the deficiencies of using a single semiconductor and shed new light on the design of advanced photocatalysts with high performance [66,67,68,69,70,71,72,73,74,75,76].

**Figure 1:**
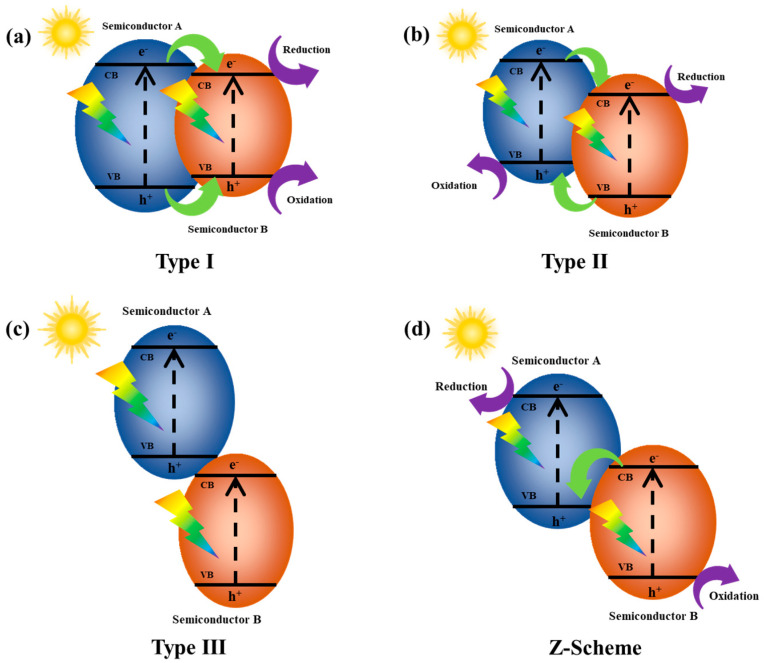
Diagram of electron-hole pair separation of (**a**) Type I; (**b**) Type II; (**c**) Type III; (**d**) Z-Scheme heterojunction [77].

Generally, the heterojunctions are classified into three types according to the relationship between the adjacent semiconductor bands: Type I, Type II, and Type III (Figure 1). Among them, the Type II heterojunctions are considered more favorable for the separation of photogenerated electron–hole pairs (Figure 1b) [77]. The photogenerated electrons migrate to the lower-level sites of the conduction band (CB) and, at the same time, holes accumulated in the valence band (VB) of the semiconductor with higher level, leading to the spatial separation of electrons and holes. This greatly reduces the recombination of charges and increases the lifetime of the charge carriers. By comparison, electrons and holes accumulate at the interfaces in Type I heterojunctions (Figure 1a). In Type III heterojunctions, there is no overlap of bands between the adjacent semiconductors (Figure 1c). This hinders the separation of charge carriers and makes them less favorable for the subsequent photocatalytic reactions.

Type II heterojunctions have attracted great attention due to their effective and efficient separation of charge carriers. A multi-interface Mn_3_O_4_@ZnO/TiO_2_ material with Type II heterojunctions was designed and prepared by alternating the growth of ZnO and TiO_2_ [78]. The heterostructured materials exhibited improved light absorption, expanded contact areas, and broadened charge transfer paths. This provides more charge transfer paths and reaction areas for selective denitrification. Similarly, Type II heterojunctions were also constructed between Cs_3_Bi_2_Br_9_ and TiO_2_ using a simple solvent method, where the built-in electric fields enhanced the local electric field intensity and facilitated the separation of the photogenerated charge carriers [79]. However, the charge carriers migrated through Type II heterojunctions with reduced redox potentials although their charge recombination was inhibited, which resulted in lower redox capabilities and limited applications. Therefore, balancing the charge separation efficiency and redox ability is crucial for the design of future photocatalysts with Type II heterostructures.

The Z-Scheme heterojunction was first proposed by Bard [80] in 1979, who simulated the natural photosynthesis system, aiming to improve the weakened redox capability of Type II heterojunctions [45]. In Type-II heterojunctions, photogenerated carriers transfer between the band edges, in which oxidation and reduction reactions occur at the VB of semiconductor A and the CB of semiconductor B, respectively. In contrast, in Z-scheme heterojunctions, electrons tend to migrate from the CB of semiconductor B to the VB of semiconductor A. The Z-Scheme heterojunction features a stepped band structure (Figure 1d), in which electrons are endowed with a strong reduction capability (electrons in the CB of semiconductor A) and holes are endowed with a strong oxidation capability (holes in the VB of semiconductor B) upon light irradiation. Bai et al. [81] constructed Z-Scheme heterojunctions in Co_3_O_4_/Cu_2_O hybrids, where the interface of Co_3_O_4_/Cu_2_O allowed for electrons to transfer from the CB of Co_3_O_4_ to the VB of Cu_2_O. Moreover, the Cu(I) in Cu_2_O favored the formation of the critical *CHO intermediates [82,83] and thus enhanced the reduction capability of the photogenerated electrons. The interfacial potential of the Co_3_O_4_/Cu_2_O photocatalyst increased under near-infrared light irradiation, which promoted photothermal conversion and provided a high-temperature environment for the photocatalytic reactions. Inspired by the innovative design of Z-Scheme heterojunctions of Co_3_O_4_/Cu_2_O materials, the Cu_2_O/TiO_2_ system was further explored with the aim of building an efficient and stable photocatalytic platform. Cu_2_O, possessing unique Cu(I) valence states, serves as an electron donor and provides key active sites for the subsequent catalytic reactions. TiO_2_’s stable structure and wide band gap are another important part of the Z-Scheme heterostructures, allowing to capture photons effectively and generate excited electron–hole pairs.

**Figure 2:**
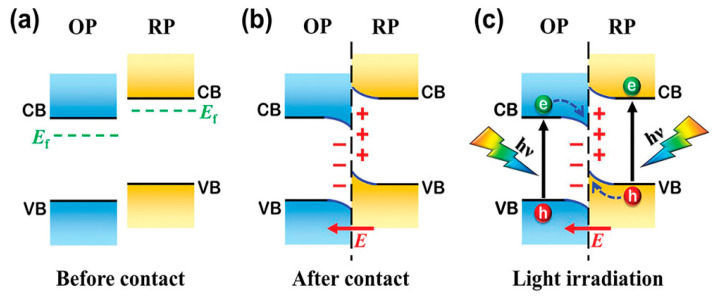
Charge-transfer processes in an S-Scheme heterojunction (**a**) before contact and (**b**) after contact; and (**c**) photogenerated charge carrier transfer under light irradiation [84].

Research on the Z-Scheme heterojunctions has achieved significant progress. However, the Z-Scheme heterojunction does not provide the Fermi level or band-bending information. Yu et al. proposed an S-Scheme heterojunction based on the direct Z-Scheme heterojunction. The S-Scheme heterojunction is composed of an oxidation photocatalyst (OP) and reduction photocatalyst (RP), where the valence band position and Fermi level of the RP (electron retention for reduction) should be higher than the OP (hole retention for oxidation). Specifically, when the RP and OP make contact, the electrons in the higher Fermi level of RP drift into the lower Fermi level of OP at the interface (Figure 2). As a result, the RP side loses electrons and becomes positively charged, while the OP side receives electrons and becomes negatively charged. In addition, an embedded electric field from RP to OP is formed when the band bends up or down at the interface, respectively [84]. Light irradiation drives the transfer of photogenerated electrons from the CB of OP to the VB of RP. In addition, the coulomb attraction and band-bending between the electrons in the OP and the holes in the RP also favor this charge transfer. Currently, Type II and Z-Scheme heterojunctions are the most extensively investigated.

### 2.2. Cu_2_O/TiO_2_ Type II Heterojunction

**Figure 3:**
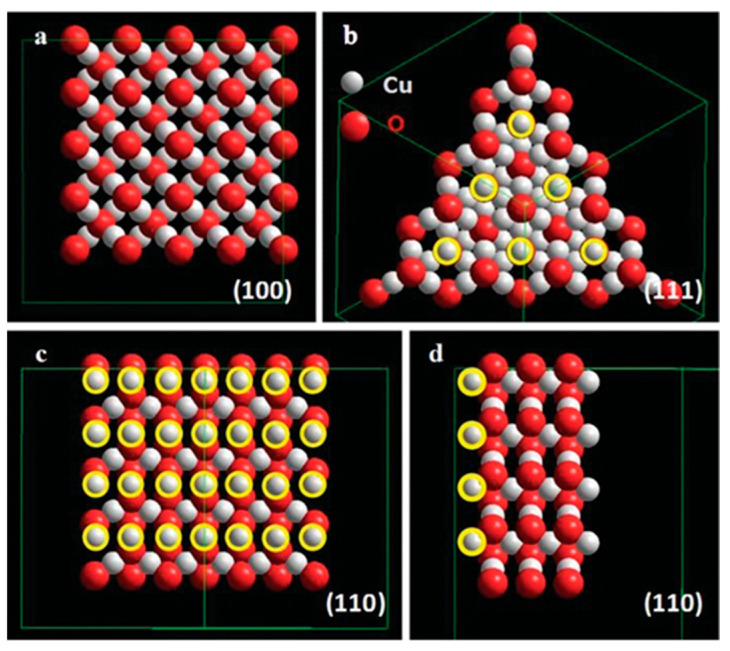
Crystal structures of Cu_2_O oriented to show the (**a**) (100); (**b**) (111); (**c**) (110); and (**d**) (110) planes. Surface Cu atoms on the (110) and (111) faces are shown with yellow circles [87].

Cuprous oxide (Cu_2_O) is an important p-type semiconductor with a bandgap of ~2.17 eV. It has been widely used in catalytic oxidation reactions, organic synthesis, photocatalysis, and photoelectrocatalysis [85]. Cu_2_O nanocrystals have three basic geometries—cubic, octahedral, and rhombic dodecahedral—formed by (100), (111), and (110) crystal planes, respectively. The (110) plane, in which all Cu atoms are exposed, provides numerous active sites for catalytic reactions. For the (100) plane, there are no exposed Cu atoms [86], while the (111) plane has partially exposed Cu atoms located in the sub-surface (Figure 3). This structural difference results in varied catalytic activities for different Cu_2_O geometries.

**Figure 4:**
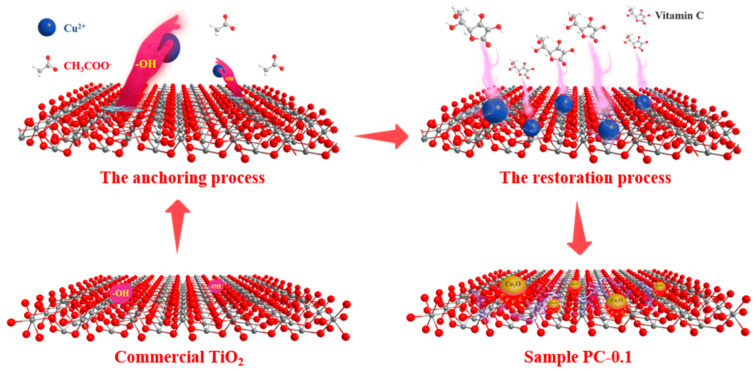
Schematic diagram of the formation mechanism of Cu_2_O quantum dots on a commercial TiO_2_ surface [89].

However, none of the Cu_2_O geometries can independently drive photocatalytic reactions due to their easy oxidation or reduction via photogenerated charges, which limits their applications. Therefore, Cu_2_O/TiO_2_ heterostructures were constructed to enhance their stability and photocatalytic efficiency. For instance, Xu et al. [88] synthesized Cu_2_O octahedra with (110) and (100) crystal faces and combined them with TiO_2_ to form Type II heterojunctions. The hybrid materials showed an excellent photocatalytic performance in the degradation of methyl orange. Hu and his coauthors [89] successfully loaded Cu_2_O quantum dots (QDs) onto commercial TiO_2_ (P25) nanoparticles. The results suggested that hydroxyl (-OH) groups of P25 played a critical role in anchoring cuprous ions, which induced the in situ generation of Cu_2_O QDs (Figure 4). Then, QDs are uniformly dispersed on the surface of P25, which significantly extends its light absorption to the visible region and enhances the separation efficiency of the photogenerated carriers and the photoreduction capability. Type II heterojunctions were formed between Cu_2_O QDs and P25, which resulted in the effective separation and transfer of electrons and improved photocatalytic activity. In hydrogen production tests, the hydrogen evolution rate over the Cu_2_O/TiO_2_ catalysts was 72 times higher than that of the single P25, showing excellent photocatalytic stability.

### 2.3. Cu_2_O/TiO_2_ Z-Scheme Heterojunction

**Figure 5:**
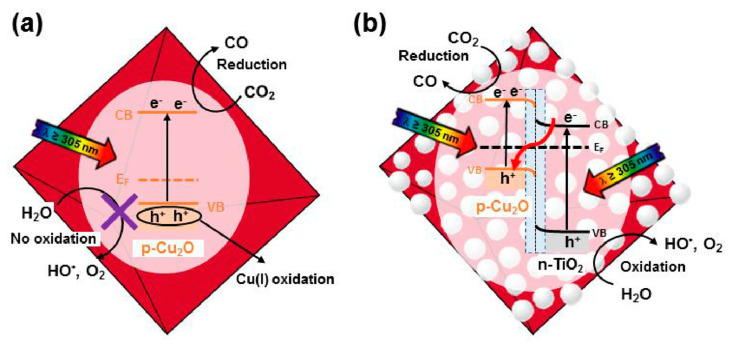
Scheme of the proposed mechanism to account for the CO_2_ reduction induced by UV-vis irradiation (λ ≥ 305 nm) of (**a**) octahedral Cu_2_O and a (**b**) Cu_2_O/TiO_2_ composite [90].

Heterostructures consisting of octahedral Cu_2_O covered with TiO_2_ nanoparticles were synthesized as photocatalysts [90]. The photoreduction rate of CO_2_ under visible light irradiation was about four times higher than that of pure octahedral Cu_2_O. The relative content of Cu(II) remained stable in Cu_2_O/TiO_2_ under illumination, as shown through a comparison of the XPS spectra of Cu_2_O/TiO_2_ and pure Cu_2_O. In contrast, pure Cu_2_O showed an increased Cu(II) content, indicating that the introduction of TiO_2_ can protect Cu_2_O from photocorrosion and confirm the formation of a Z-Scheme heterojunction between Cu_2_O and TiO_2_. Additionally, hydroxyl radicals generated from water oxidation were detected, which was a critical step in the Z-Scheme mechanism (Figure 5). The Cu_2_O/TiO_2_ materials exhibit great potential for use in photocatalysis, especially in environmental and energy-related fields.

More research on heterostructured Cu_2_O/TiO_2_ materials has been reported in recent years. For example, Lv et al. [91] loaded Cu_2_O onto TiO_2_ nanomaterials, and Z-Scheme heterojunctions were formed and successfully used in hydrogen production through the photolysis of seawater. The photocatalytic efficiency of hydrogen production was significantly enhanced by using Cu_2_O/TiO_2_ composites as photocatalysts [92]. These results indicate that the construction of Cu_2_O/TiO_2_ heterojunctions addresses the instability challenges of Cu_2_O to some extent and provides new possibilities for achieving more efficient photocatalytic systems. Thus, an in-depth exploration of the formation mechanism of Cu_2_O/TiO_2_ heterojunctions, interface engineering, and catalytic performance optimization is crucial for the design of novel photocatalytic materials and advanced photocatalytic technology.

### 2.4. Methods for the Construction of Cu_2_O/TiO_2_ Heterojunctions

Recently, various methods, including hydrothermal synthesis, impregnation reduction, electrochemical deposition, and liquid-phase reduction, have been developed for the construction of Cu_2_O/TiO_2_ heterojunctions, and each of them has unique advantages and distinct preparation conditions.

#### 2.4.1. Hydrothermal Synthesis

Cu_2_O/TiO_2_ heterojunctions are synthesized in aqueous environments at a high temperature and high pressure. During the hydrothermal processes, titanium sources (e.g., titanium tetrabutoxide) and copper sources (e.g., copper salts) react at high temperatures and generate Cu_2_O/TiO_2_ nanostructures. The morphology and size of the Cu_2_O/TiO_2_ heterostructures can be modulated by changing the reaction temperature, the reaction time, and the concentration of precursors. Using this approach, well-crystallized and uniformly distributed heterojunctions can be mass-produced at low costs. When using Cu(CH_3_COO)_2_ as the precursor and dispersing TiO_2_ nanoparticles in a Cu^2+^ solution before transferring them to a high-pressure reactor, Lv et al. [91] produced tightly bound Cu_2_O/TiO_2_ photocatalysts. However, it is necessary to obtain a balance between cost and energy efficiency in industrial production because high levels of energy consumption and long reaction times are required in typical hydrothermal synthesis.

#### 2.4.2. Impregnation–Reduction Method

In this method, a carrier material containing reducing agents is impregnated into a metal salt solution in which a reduction reaction is promoted, and metal or metal oxide is formed on the surface of the carrier material. TiO_2_ nanotubes were immersed in an anhydrous ethanol solution of Cu(NO_3_)_2_·3H_2_O for 24 h; then, the photocatalytic reduction in Cu^2+^ was induced to form Cu@Cu_2_O core-shell nanoparticles under UV irradiation [93]. Thus, Cu_2_O/Cu/TiO_2_ heterojunctions were generated. The impregnation-reduction method is simple and cost-effective, and the morphology and distribution of Cu_2_O nanocrystals can be well-controlled by changing the impregnation time, temperature, and reducing agent concentration, allowing for wide applicability.

#### 2.4.3. Electrochemical Deposition

Electrochemical deposition is a liquid-phase preparation method. Using this method, the desired materials can be deposited on the electrode surface through electrochemical reduction reactions. TiO_2_ films were first prepared via hydrothermal synthesis; then, TiO_2_-Cu_2_O heterojunctions were constructed using a constant-potential electrodeposition method in an electrolyte containing CuSO_4_·5H_2_O and lactic acid [94]. The electrochemical deposition method has the advantages of simplicity, low cost, ease of control, and room-temperature operation, which allows for precise control over the material film’s thickness and composition. However, defects and impurities may be introduced during the electrochemical deposition process, and thus have negative effects on the migration of electrons and holes.

## 3. Mechanism of Charge Transfer Across the Cu_2_O/TiO_2_ Heterojunctions

### 3.1. Type II Electron Transfer

**Figure 6:**
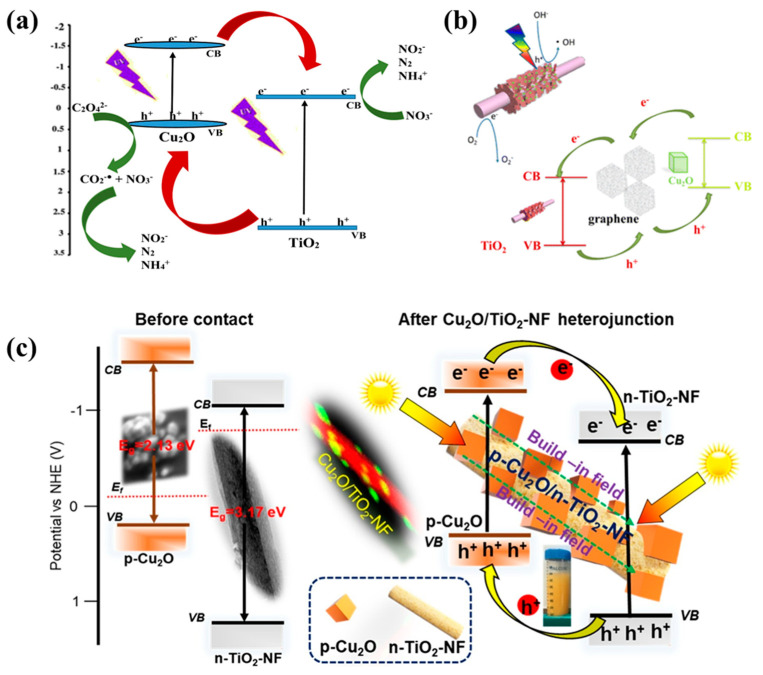
(**a**) Proposed mechanism for the simultaneous photocatalytic removal of nitrate ions and oxalic acid from the Cu_2_O/TiO_2_ photocatalyst [97]; (**b**) the proposed photocatalytic mechanisms of TiO_2_ NSAs/G/Cu_2_O [98]; (**c**) band alignment energy diagram of Cu_2_O, TiO_2_-NF before and after contact. (*CB*: conduction band, *VB*: valence band, *E_f_*: Fermi level) [99].

In Type II heterojunctions, photogenerated charge carriers transfer between the adjacent band edges, with oxidation occurring at the highest valence band and reduction at the lowest conduction band of the heterojunctions [95]. Therefore, the catalytic performance is significantly influenced by the bandgap, and the positions of VB and CB [96]. Cu_2_O/TiO_2_ and activated carbon (AC) composites were used as photocatalysts to remove nitrate and oxalate ions from water [97]. The results indicated that the incorporation of Cu_2_O effectively broadened the light response of TiO_2_ and simultaneously accelerated the removal of nitrate ions and oxalate ions. The enhancement in the photocatalytic activity was attributed to the formation of *p-n* heterojunctions between Cu_2_O and TiO_2_ nanocrystals, which inhibited the recombination of photogenerated electrons and holes. Under light irradiation, electrons transfer from the conduction band of Cu_2_O to the conduction band of TiO_2_, while holes migrate from the valence band of TiO_2_ to the valence band of Cu_2_O, promoting nitrate ions’ reduction and oxalate ions’ oxidation (Figure 6a). The introduction of AC in the photocatalytic system improved the N_2_ selectivity but decreased the photocatalytic efficiency, suggesting that N_2_ generation can be optimized by controlling the oxalate consumption rates.

Novel hybrid photocatalysts (TiO_2_ NSAs/G/Cu_2_O) were prepared by introducing graphene (G) into TiO_2_ nanosheet array (NSAs)/Cu_2_O composites (Figure 6b), where graphene acts as an electron trap to inhibit the recombination of electrons and holes [98]. Additionally, TiO_2_ nanosheets provided favorable transport channels for charge carriers, further reducing the probability of their recombination. The three-dimensional photocatalysts were formed on carbon cloth, which increased the area of light exposure and provided more active sites for reactant adsorption, enhancing their photocatalytic performance. The three-dimensional structure allowed numerous photons to penetrate deeply into the photocatalysts, improving the efficiency of light utilization. When both TiO_2_ and Cu_2_O are excited under light illumination, the holes in the valence band of TiO_2_ could migrate to the valence band of Cu_2_O because the VB of TiO_2_ is more positive than that of Cu_2_O. Similarly, electrons could transfer from the conduction band of Cu_2_O to TiO_2_ due to the higher conduction band potential of Cu_2_O.

Sekar et al. [99] reported an efficient heterostructured photocatalyst for H_2_ generation and sulfamethoxazole (SMX) oxidative degradation by using cubic Cu_2_O nanoparticles to modify TiO_2_ nanofibers (Figure 6c). The results confirmed that the heterojunctions between Cu_2_O and TiO_2_ were conductive to charge transfer and active sites were formed; thus, their photocatalytic performance was enhanced. Additionally, band structure analysis showed that the formation of the Cu_2_O/TiO_2_ heterojunction led to a shift in the energy band and the creation of space charge regions, which promoted electron–hole separation, extended the lifetime of charge carriers [100], and thus significantly improved photocatalytic activity under visible light.

### 3.2. Z-Scheme Electron Transfer

Z-Scheme heterojunctions have similar band alignments to Type II heterojunctions but different transport mechanisms for charge carriers. Unlike Type II heterojunctions, in which the photogenerated electrons transfer between the conduction bands, in Z-scheme heterojunctions, electrons tend to migrate from the CB of semiconductor B to the VB of semiconductor A. This allows reduction reactions to occur at the CB of the semiconductor with higher reduction potential while the oxidation reaction occurs at the VB of the semiconductor with more positive potential, inhibiting the recombination of charge carriers but not at the cost of their redox ability. Z-Scheme heterojunctions are divided into indirect and direct forms of contact according to their band contact patterns.

#### 3.2.1. Indirect Contact

**Figure 7:**
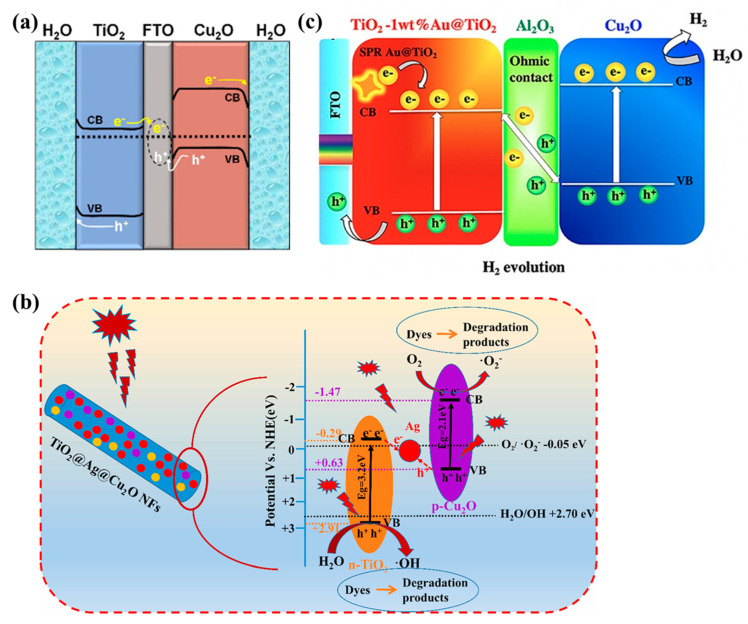
(**a**) Energy band structure diagram of TiO_2_/Cu_2_O@FTO panel system [101]; (**b**) mechanism for the photocatalytic degradation of dyes using TiO_2_@Ag@Cu_2_O NFs under simulated sunlight irradiation [102]; (**c**) a schematic visualization of a bifunctional PEC system with a direct Z-scheme mechanism of TiO_2_-1 wt% Au@TiO_2_/Al_2_O_3_/Cu_2_O photoelectrodes in an H-cell type reactor [103].

Since Bard proposed liquid-phase Z-Scheme heterojunctions in 1979, researchers have explored other electron transfer media, in addition to liquid redox mediators. Solid-state Z-Scheme heterojunctions use solid conductors as the electron media between two semiconductors. For example, a thin-film photocatalyst was constructed by alternately patterning TiO_2_ and Cu_2_O stripes on a conductive Fluorine-doped Tin Oxide (FTO) substrate for water-splitting (Figure 7a) [101]. The Z-Scheme charge transfer mechanism between TiO_2_ and Cu_2_O stripes through the FTO substrate was confirmed via tracking the photogenerated charges using a photochemical deposition method, and it was found that the optimal transfer distance is about 5 µm. The results indicated that the Z-Scheme charge transfer exhibited a superior performance in terms of water-splitting and provided new insights for the design of efficient heterostructures.

Metals can also be used as electron transfer media, in addition to FTO conductive glass. For instance, TiO_2_@Ag@Cu_2_O nanofibers with Z-Scheme heterojunctions were obtained via electrospinning [102]. As photocatalysts, the heterostructured materials showed a 99% degradation rate of methylene blue under visible light within 2 h. The efficient use of sunlight and enhanced charge separation rates were fulfilled owing to the redshift in light absorption caused by the Cu_2_O and Ag doping-induced surface plasmon resonance effect. Electrochemical tests showed that the Z-Scheme heterojunction was formed in TiO_2_@Ag@Cu_2_O hybrid (Figure 7b), which endowed it with a higher photocurrent density compared with TiO_2_@Cu_2_O nanofibers (3.78 times) and pure TiO_2_ nanofibers (47.6 times), indicating improved visible light absorption and charge separation efficiency.

Due to the high cost of Z-Scheme heterojunctions that use conductive metals as intermediate layers, researchers have explored the use of other semiconductor materials as the electron transfer media. A multi-layer TiO_2_-1 wt% Au@TiO_2_/Al_2_O_3_/Cu_2_O photocatalyst (Figure 7c) was prepared by dispersing and partially embedding Cu_2_O nanoparticles on TiO_2_ nanofibers [103]. The narrow bandgap of Cu_2_O enables it to absorb visible light, and the Al_2_O_3_ ultrathin layer acts as a barrier against the rapid recombination of electrons and holes, promoting charge separation. The electrons collected in Cu_2_O contributed to the reduction in water during hydrogen production, while holes in TiO_2_ oxidized organic pollutants such as humic acid. Humic acid acted as an electron donor, capturing holes generated during the photocatalysis process, suppressing the charge recombination, and enhancing the photodegradation efficiency. Compared to liquid-phase Z-Scheme heterojunctions, solid Z-Scheme heterojunctions avoid certain reverse reactions, favor the recovery of catalysts, and are suitable for both liquid- and gas-phase reactions. However, solid Z-Scheme photocatalysts often use expensive electron conductors, which limits their further application.

#### 3.2.2. Direct Contact

The incorporation of intermediate conductors in indirect contact-type Z-Scheme heterojunctions prevents the charges from directly transferring across the interfaces, which affects the transfer efficiency of the charge carriers and the photoelectric conversion efficiency to some extent. Therefore, researchers developed direct-contact Z-Scheme heterojunctions to avoid these drawbacks and achieve more efficient light utilization. A Z-Scheme heterostructured photocatalyst composed of two tightly contacting semiconductors was shown to remarkably extend the lifetime of charge carries [104]. This sheds new light on the design of photocatalysts with direct Z-Scheme heterojunctions. In such cases, electrons and holes can directly transfer from one material to another since there are no intermediates and no spatial isolation between different semiconductors. Furthermore, direct contact also results in significant band-bending at the heterojunction and generates internal solid electric fields. This is conducive to charge separation and migration, allowing efficient spatial separation to be achieved while retaining strong redox capabilities.

**Figure 8:**
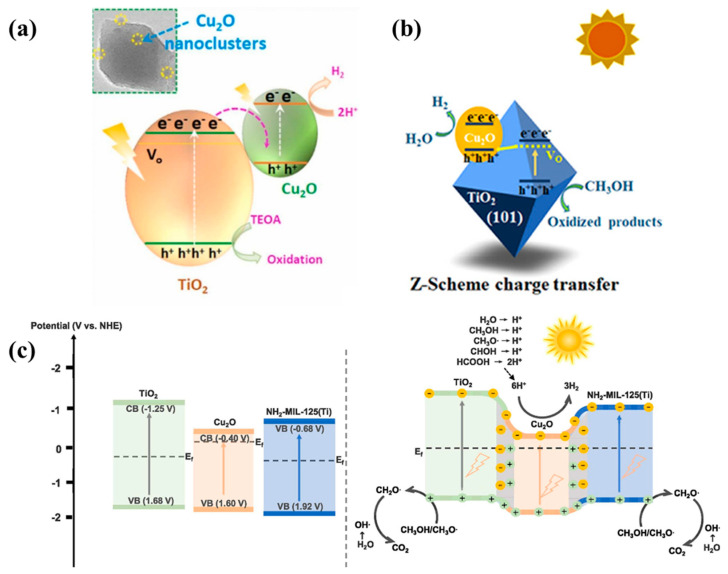
(**a**) Schematic diagram of the electron transport mechanism of a Cu_2_O/TiO_2_ nanocomposite photocatalyst [105]; (**b**) schematic diagram of interfacial charge transfer in Cu_2_O/T1 heterostructures [106]; (**c**) potential energy diagram of Cu_2_O, NH_2_-MIL-125(Ti), and TiO_2_ before contact and after contact to catalyze the evolution of photothermal catalytic H_2_ [107].

A Z-Scheme heterojunction was successfully synthesized by anchoring size-controlled Cu_2_O nanoclusters on the surfaces of TiO_2_ using the DES method [105]. Electric fields were generated between the interfaces of Cu_2_O and TiO_2_ through band-bending. This promotes charge transfer and inhibits the recombination of electrons and holes, facilitating the migration of electrons to the conduction band of Cu_2_O and the migration of holes to the valence band of TiO_2_ (Figure 8a). The enhanced efficiency of charge separation and transfer is crucial for improving the efficiency of hydrogen evolution. Additionally, the increase in surface-active sites and reduced charge transfer resistance guarantee easier carrier migration, and thus stimulate the photocatalytic reactions. This research provides a new strategy for developing efficient and green photocatalysts for hydrogen production.

The photo-corrosion of Cu_2_O presents a great challenge to its photocatalytic applications. Oxygen vacancies were first introduced on (1 0 1) faces of TiO_2_, and then Cu_2_O/T1-V_O_ materials were obtained through the photodeposition of Cu_2_O on the surface of TiO_2_ [106]. The creation of oxygen vacancies enhanced the visible light absorption of TiO_2_ and promoted the separation of photogenerated charges. Moreover, the defects (oxygen vacancies) acted as electron-trapping centers, facilitated the rapid electron transfer from Cu_2_O to TiO_2,_ and inhibited charge recombination (Figure 8b). The direct recombination of electrons at the CB of TiO_2_ with holes at the VB of Cu_2_O effectively removed the oxidative holes of Cu_2_O. This could inhibit the photo-corrosion of Cu_2_O and achieve Z-Scheme charge transfer between TiO_2_ and Cu_2_O. Density functional theory (DFT) calculation and experimental investigation demonstrated the significant effects of crystal faces and defects in the photocatalysts on interfacial charge transfer. Electrons can be more effectively injected into 3D orbitals of Cu_2_O from TiO_2_ with oxygen vacancies created in the (1 0 1) faces, forming stable Cu_2_O and thus enhancing their photocatalytic activity. Therefore, the photo-corrosion of Cu_2_O can be avoided and the photocatalytic efficiency of water splitting can be improved through the Z-Scheme charge transfer mechanism.

Dual Z-Scheme heterojunctions composed of ternary materials have also been developed, in addition to common Z-Scheme heterojunctions made up of binary materials, where the three materials make direct contact, without other mediators, for charge transfer. This structure enhances the separation efficiency of electrons and holes. A photosensitive and thermosensitive photocatalyst (Cu_2_O/NH_2_-MIL-125@TiO_2_) was designed and prepared through a controlled in situ derivation process, where Cu_2_O/NH_2_-MIL-125@TiO_2_ composites were synthesized by the introduction of a TiO_2_ layer into NH_2_-MIL-125(Ti) and the subsequent encapsulation of Cu_2_O [107]. The internal electric field generated by Cu_2_O had a more negative Fermi level compared to NH_2_-MIL-125(Ti) and TiO_2_, and thus facilitated the transfer of electrons from TiO_2_ and NH_2_-MIL-125(Ti) to Cu_2_O (Figure 8c). Under light irradiation, the photogenerated electrons reduced the number of water molecules to produce hydrogen, while the holes oxidized methanol or water molecules to form hydroxyl radicals, which further reacted with methanol to form intermediates (e.g., CH_2_O·). The intermediates were converted to carbon dioxide (CO_2_) and more hydrogen through oxidation. The incorporation of Cu_2_O in the dual Z-Scheme heterostructures and the synergy effect with NH_2_-MIL-125@TiO_2_ significantly improved their photocatalytic efficiency.

## 4. Photocatalytic Applications of Cu_2_O/TiO_2_ Heterojunctions

### 4.1. Photocatalytic Hydrogen Production

Hydrogen has become one of the most preferred alternative fuels owing to its high calorific value and stability, as well as its producing zero greenhouse gas emissions. However, most hydrogen is produced from fossil resources at present. The use of photocatalytic water splitting for hydrogen production has attracted extensive attention due to its energy conservation, environmentally friendly nature, and low cost. The design and preparation of efficient and stable photocatalysts for hydrogen production form a fast-growing research field [108,109,110,111].

**Figure 9:**
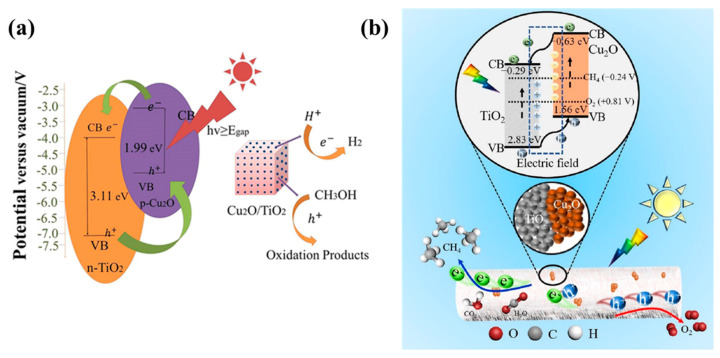
(**a**) Mechanism for the Cu_2_O/TiO_2_ catalyzed water-splitting reaction [114]; (**b**) schematic illustration of the photocatalytic CO_2_ reduction in the TiO_2_/Cu_2_O composite [96].

A hybrid Cu_2_O/N-TiO_2_ photocatalyst with enhanced electron–hole separation efficiency and quantum yield was prepared through the formation of *p-n* heterojunctions [112]. The hybrid photocatalysts showed a high hydrogen production rate of 7139.02 µmol g^−1^ h^−1^ in methanol solution under visible light irradiation, suggesting their excellent photocatalytic activity and photostability. A series of Cu_2_O/TiO_2_ photocatalysts with different Cu_2_O molar fractions were prepared via a simple modified ethanol induction method [113]. Combining n-type TiO_2_ and p-type Cu_2_O significantly enhanced their photocatalytic activity. Specifically, the formation of Cu_2_O/TiO_2_ heterojunctions improved their visible light absorption, effectively suppressed the charge recombination, enhanced the interfacial charge transfer, and, at the same time, provided abundant active sites for reaction. The highest H_2_ production rate compared to the prepared Cu_2_O/TiO_2_ photocatalysts reached 2048.25 µmol g^−1^ h^−1^, 14.48 times higher than that of pure P25, indicating that the optimization of Cu_2_O content can significantly enhance the photocatalytic activity of hybrid Cu_2_O/TiO_2_ photocatalysts. The effects of scavengers (methanol, anhydrous ethanol, ethylene glycol, and glycerol) on photocatalytic H_2_ evolution rates were also reported. The results showed that methanol is an optimal scavenger for hydrogen evolution while anhydrous ethanol resulted in a lower conversion efficiency. Similarly, a heterostructured material based on cubic Cu_2_O and TiO_2_ nanoparticles was successfully synthesized via a simple hydrothermal method [114], where TiO_2_ nanoparticles were uniformly distributed on the surface of Cu_2_O cubic crystals. The morphology of the hybrid materials and their photophysical and photoelectrochemical performances can be effectively adjusted by controlling the amount of TiO_2_ (Figure 9a).

Generally, Cu_2_O/TiO_2_ hybrids, characterized by their *p-n* heterojunctions, have demonstrated remarkable efficiency in photocatalysis. Their potential in photocatalytic hydrogen production is notably substantial, as evidenced by the various studies summarized in Table 1. The Cu_2_O/TiO_2_ heterojunction’s promising application in sustainable energy is shown by its ability to efficiently convert light energy to chemical energy, particularly in hydrogen production. However, certain challenges such as the high recombination rates of photogenerated charge carriers, their unsatisfactory stability, and their insufficient active sites limit their further application. Future attention should be focused on optimizing the heterojunction interfaces, enhancing the stability of materials, improving light absorption, and exploring their scalability and the feasibility of commercialization. These efforts are expected to overcome the existing challenges and improve the efficiency of hydrogen evolution using Cu_2_O/TiO_2_ photocatalysts.

### 4.2. Photocatalytic CO_2_ Reduction

In recent years, the increase in atmospheric CO_2_ concentrations has attracted great attention and it is recognized as a primary greenhouse gas. The utilization of solar energy to reduce CO_2_ into high-value chemicals or fuels is an attractive [120,121,122,123,124,125] and environmentally friendly approach [126,127,128] to address the present challenges of global warming and energy shortages.

In this context, Cu_2_O/TiO_2_ heterojunctions were synthesized in order to expand their light absorption and obtain the optimized separation of electron–hole pairs [129,130]. For instance, Schreier et al. [131] reported a means of covalently anchoring molecular catalysts on the surface of Cu_2_O photoelectrodes for light-driven CO_2_ reductions. The photocurrent density was significantly enhanced, and high CO selectivity was achieved by depositing nanostructured TiO_2_ layers on Cu_2_O. This approach reduced catalyst usage and showed a high utilization of solar light energy, providing a new idea for the design of efficient solar fuel devices. However, the activity of the catalyst decreased with the operation time, highlighting the requirement for stability and regeneration strategies in future catalysts.

A mesoporous TiO_2_ nanorod composite loaded with Cu_2_O nanoparticles was prepared via chemical reduction to enhance the photocatalytic reduction efficiency of CO_2_ [96]. The photochemical and electrochemical measurements showed that the TiO_2_/Cu_2_O-15% sample exhibited stronger photocurrents and lower charge transfer resistance owing to the built-in electric field, which accelerates its efficient charge separation and migration capabilities and endows it with improved photocatalytic efficiency (Figure 9b). The production rate of methane compared to the TiO_2_/Cu_2_O-15% photocatalysts was significantly higher than that of pure TiO_2_ and Cu_2_O. This *p-n* junction of the hybrids favored the built-in electric field, where electrons were easily transferred from Cu_2_O to TiO_2_ while holes were transferred from TiO_2_ to Cu_2_O, improving the photocatalytic efficiency of CO_2_ to CH_4_.

Cu_2_O/TiO_2_ heterojunctions hold great potential in photocatalytic CO_2_ reductions (Table 2). However, some critical issues, including the separation efficiency of photogenerated charges, and the adsorption and activation of CO_2_, should be improved, while the cost of photocatalysts should be reduced to adapt to large-scale production. Future research should focus on the understanding of the charge transfer mechanisms across the interfaces, the optimization of photocatalytic reaction conditions, and the large-scale preparation of photocatalysts. These efforts are expected to advance the commercialization of Cu_2_O/TiO_2_ photocatalysts in CO_2_ reductions, helping to address global climate change and obtain high-value chemicals or fuels.

### 4.3. Pollutant Degradation

Large quantities of organic pollutants have infiltrated our ecosystems and exerted unprecedented pressure on the environment over the past decades of rapid urbanization and population growth [135,136,137,138,139]. Due to their different toxicity levels and resistance to oxidation, organic pollutants pose significant threats to human health. Various treatments, including adsorption [140,141], biological oxidation [142], chemical treatments, and photodegradation [143,144], have been applied to remove the harmful pollutants. Among these methods, the photocatalytic degradation of contaminants has shown high selectivity for organic pollutants, reducing the damage to ecosystems and the cost of treatments.

A 3D composite photocatalyst was fabricated by dispersing Cu_2_O nanocrystals (NCs) onto TiO_2_ photonic crystal (PC) frameworks [145]. This composite exhibited enhanced photocatalytic activity in the degradation of rhodamine B and bisphenol A under UV and visible light. This is attributed to the synergistic light absorption effect of the TiO_2_/Cu_2_O Type II heterojunctions and the photonic effect of TiO_2_ PC, amplifying the light absorption of Cu_2_O NCs. Additionally, the recombination of electron–hole pairs was effectively inhibited and quantum efficiency was remarkably improved owing to their Type II heterojunctions. The Cu_2_O NCs/TiO_2_ PC photocatalysts were synthesized and applied in the degradation of p-nitrophenol (PNP). The formation of Type II heterojunctions between Cu_2_O NCs and TiO_2_ PC enhanced light absorption, the charge separation efficiency, and the final conversion of PNP [146].

Cu_2_O/TiO_2_ photocatalytic systems exhibit great potential in pollutant degradation due to their special band structures and good photocatalytic performance (Table 3). However, challenges such as their insufficient stability under light irradiation, the high recombination rate of photogenerated carriers, and their limited utilization of visible light still exist. Future attention should be focused on improving the stability of materials, optimizing their interface structures, enhancing visible light absorption, exploring reaction mechanisms, and scaling up applications to advance the application of the photocatalytic degradation of pollutants in environmental governance.

### 4.4. Selective Synthesis

Selective synthesis stems from the pursuit of product selectivity in chemical synthesis. Methods to enhance the selectivity of target products in synthesis processes have been widely explored, accompanied by the development of the chemical industry and the growing demand for fine chemicals. Among the approaches to improve the yields of target products, light-driven selective synthesis has attracted special attention owing to its high efficiency and environmentally friendly nature. Target products with high purity could be obtained through the design of novel catalysts, selection of reaction paths, and optimization of reaction conditions, showing great potential in various fields [152,153,154,155,156].

**Figure 10:**
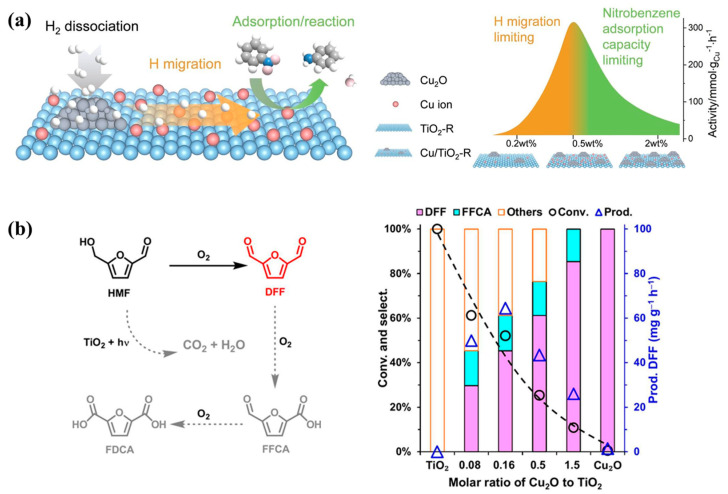
(**a**) Schematic of nitrobenzene hydrogenation on Cu/TiO_2_-R and rate-limiting step changes in nitrobenzene hydrogenation on xCu/TiO_2_-R (x = 0.2–2 wt%) and the proposed colonies of H_2_ dissociation and nitrobenzene adsorption/hydrogenation sites [157]; (**b**) the main reaction pathways and photocatalytic oxidation of HMF on TiO_2_, Cu_2_O, and (Cu_2_O)_x_‖TiO_2_ [158].

Rutile TiO_2_-based materials (Cu/TiO_2_-R), with supported Cu_2_O nanoparticle sites and dispersed single Cu^+^ ion sites, were prepared via direct H_2_/Ar reduction [157]. The catalyst exhibited excellent selectivity and stability for aromatic amines in the hydrogenation of nitroaromatic hydrocarbons. Nitrobenzene primarily adsorbed on highly dispersed Cu^+^ sites on TiO_2_-R surfaces. The dissociated hydrogen migrated from Cu_2_O nanoparticles to TiO_2_-R surfaces and hydrogenated the adsorbed nitrobenzene (Figure 10a). The optimal performance was obtained for the 0.5Cu/TiO_2_-R catalyst due to the synergistic effects of single Cu^+^ sites and Cu_2_O nanoparticles. Compared to 0.5Cu/TiO_2_-R, the 2Cu/TiO_2_-R catalyst had more H_2_ dissociation sites and shorter Cu^+^ distances for H migration but fewer nitrobenzene adsorption sites. This study not only differentiated the roles of Cu_2_O nanoparticles and dispersed Cu^+^ ions during hydrogen dissociation and nitrobenzene hydrogenation but also revealed the role of Cu^+^ in hydrogen migration, providing new insights into selective hydrogenation.

The selective oxidation of 5-hydroxymethylfurfural (HMF) to 2,5-furandicarboxaldehyde (DFF) represents a critical step in converting biomass-derived furan molecules into high-value-added products. The conversion efficiency of HMF to DFF and the selectivity of DFF was effectively improved by the construction of (Cu_2_O)_x_‖TiO_2_ *p-n* heterojunctions [158]. The heterojunctions suppressed the formation of hydroxyl radical on TiO_2_ surfaces and thus reduced the side reactions. The heterojunctions also promoted the activation of oxygen on Cu_2_O surfaces and the generation of singlet oxygen, and therefore accelerated the formation of DFF (Figure 10b). The construction of (Cu_2_O)_x_‖TiO_2_ *p-n* heterojunctions not only expanded the light utilized by TiO_2_ but also improved the redox capabilities of Cu_2_O, offering new pathways for the selective synthesis of furan molecules and important references for the conversion of biomass such as lignin to high-value-added products.

In photocatalysis, selective synthesis allows for precise control over photocatalytic reaction paths and products, which will improve the yield of the target product and reduce the formation of by-products. This technology not only enhances the efficiency and sustainability of photocatalytic reactions but also helps to develop new photocatalysts, and thus promotes the application and development of photocatalytic technology [159,160,161,162,163]. Therefore, selective synthesis has broad applications and is of significant value in photocatalysis.

## 5. Conclusions and Future Outlook

With the depletion of global fossil energy resources and the escalating environmental pollution issues, the development of high-powered photocatalysts that are capable of efficiently utilizing solar energy is crucial. By simulating natural photosynthesis, solar energy can be converted into chemical energy to drive chemical reactions, achieving clean energy production and degrading environmental pollutants. The construction of Cu_2_O/TiO_2_ heterojunctions not only addresses the shortcomings of TiO_2_ but also effectively mitigates the photo-corrosion issue of Cu_2_O, as well as having wide application in hydrogen evolution, CO_2_ reduction, pollutant degradation, and selective synthesis.

Cu_2_O/TiO_2_ heterojunctions exhibit great potential in photocatalysis by combining the advantages of Cu_2_O and TiO_2_. The complementary bandgaps of Cu_2_O and TiO_2_ enable a more wide-spectrum response compared to their individual use. The heterojunction interfaces promote the separation of photogenerated electron–hole pairs, extend the lifetime of charge carriers, and improve the transfer efficiency of electrons through various charge transfer paths. Additionally, Cu_2_O and TiO_2_ are abundant and cost-effective compared to other photocatalysts. However, the current research on Cu_2_O/TiO_2_ heterojunctions has revealed limitations, such as the interfacial defects formed during the heterojunction formation, which lead to increased charge recombination. Future investigations should focus on improving carrier separation efficiency and photocatalytic performance. Furthermore, the poor stability of Cu_2_O in humid or acidic environments was observed, although the formation of heterojunctions partially mitigates photocorrosion, which has adverse effects on its long-term performance and durability. Reasonable doping, adjusting the electronic and optical properties of the materials, and developing novel hybrid materials including metal nanoparticles could further enhance the light absorption and charge separation efficiency.

## Figures and Tables

**Table 1:** Comparison of Cu_2_O/TiO_2_ heterojunction photocatalytic hydrogen production.

Catalyst	Heterojunction	Sacrificial	Rate/mmol g^−1^ h^−1^	Ref.
Cu_2_O/T1-V_O_	Z-Scheme	Methyl alcohol	32.600	[106]
Cu_2_O/TiO_2_	Z-Scheme	Triethanolami	24.210	[105]
Cu_2_O/TiO_2_	Type I	Triethanolami	14.390	[115]
TiO_2_/Cu_2_O/Cu	Type II	Methyl alcohol	8.500	[116]
Cu_2_O/N-TiO_2_	Type II	Methyl alcohol	7.139	[112]
Cu_2_O/TiO_2_	Type II	Methyl alcohol	2.550	[117]
Cu/Cu_2_O/P25	Type II	Methyl alcohol	2.143	[118]
Cu_2_O/TiO_2_	Type II	Water	2.048	[113]
Cu_2_O@TiO_2_	Type II	Triethanolamine	1.180	[119]
Cu_2_O/TiO_2_	Type II	Water	0.500	[114]

**Table 2:** Comparison of the photocatalytic reduction of CO_2_ using various Cu_2_O/TiO_2_ heterojunctions.

Catalyst Name	Heterojunction	Intended Product	Productivity (µmol g^−1^ h^−1^)	Ref.
Cu^+^/TiO_2_-1	Type II	CO	43.5	[132]
Pd/Cu_2_O/TiO_2_	Type II	CH_4_	42.8	[133]
Cu^+^/TiO_2_-1	Type II	CH_4_	16.7	[132]
Cu_2_O/TiO_2_	Type II	CH_3_OH	11.77	[130]
TiO_2_/Cu_2_O	Type II	CO	10.22	[134]
Cu_2_O/TiO_2_	Z-Scheme	CO	2.11	[90]
TiO_2_/Cu_2_O	Type II	CH_4_	1.35	[96]

**Table 3:** Comparison of the photocatalytic degradation of pollutants by various Cu_2_O/TiO_2_ heterojunctions.

Catalyst Name	Heterojunction	Pollutant	Removal Rate/% (Time)	Ref.
TiO_2_/Cu_2_O/Cu	Type II	Rhodamine B	100 (2 h)	[116]
Cu-Cu_2_O-TiO_2_	Z-Scheme	Methyl Orange	96 (1 h)	[147]
TiO_2_/Cu_2_O	Type II	Methyl Orange	91 (6 h)	[148]
Cu_2_O NCs/TiO_2_ 260	Type II	Rhodamine B	90 (0.5 h)	[145]
Cu_2_O NCs/TiO_2_ 260	Type II	Bisphenol A	85 (0.5 h)	[145]
Cu_2_O/TiO_2_/Pt	Type II	Methyl Orange	80 (2 h)	[149]
Cu_2_O/TiO_2_	Type II	P-Nitrophenol	70 (4 h)	[146]
Cu_2_O-doped TiO_2_	Type II	Diclofenac	70.07 (5 h)	[150]
Cu_2_O-(rGO-TiO_2_)	Z-Scheme	2,2′4,4′-Tetrabromodiphenyl	56 (3 h)	[151]
